# Gastric-filling ultrasonography to evaluate gastric motor function in patients long-term bedridden undergoing stroke

**DOI:** 10.3389/fphys.2025.1472096

**Published:** 2025-03-04

**Authors:** Jing Xu, Xiaoxuan Li, Kaiwen Xue, Ying Xu, Ruixue Ye, Jingpu Zhao, Xuehui Fan, Linlin Shan, Yulong Wang

**Affiliations:** ^1^ Department of Rehabilitation, Shenzhen Second People’ Hospital, The First Affiliated Hospital, Shenzhen University School of Medicine, Shenzhen, Guangdong, China; ^2^ Rehabilitation Medical College, Shandong University of Traditional Chinese Medicine, Jinan, China

**Keywords:** gastric ultrasonography, gastric emptying, gastric motility, long-term bedridden, stroke patients

## Abstract

**Objectives:**

Gastric motor dysfunction is a common symptom in patients with stroke, yet lacks objective evaluation methods. This study aimed to assess the feasibility of using gastric filling ultrasonography to evaluate gastric motor function in patients undergoing stroke, and to explore the relationship between gastric ultrasound indicators and clinical features.

**Methods:**

We conducted a case-control study where all participants underwent a 60-minute ultrasound examination after consuming a 300 mL test meal. The cross-sectional area (CSA) of the gastric antrum was measured at four-time points: fasting for six to eight hours (T0), immediately after the test meal (T1), and at 30 (T30) and 60 (T60) minutes. Using CSA, we calculated the gastric emptying rate (GER) at T30 and T60 (GER30, GER60). Additionally, we measured the frequency (ACF) and amplitude (ACA) of antral contractions, as well as the motor index (MI). We compared these ultrasound parameters between the two groups and evaluated their correlation with clinical features such as bed rest time, consciousness level, albumin or hemoglobin levels.

**Results:**

We recruited 37 stroke patients and 31 healthy controls. Stroke patients exhibited lower GER compared to controls, particularly evident at T30. Additionally, stroke patients showed significantly reduced ACA, ACF, and MI at T1 and T30, with ACA being the only measure showing statistical differences at T60. Correlation analysis revealed negative associations between ACA, GER30, GER60, MI_T1_ and bed rest time. For predicting anemia, GER30 had a cut-off of 31.52 (88% specificity, 50% sensitivity), while ACA_T60_’s cut-off was 23.64 (76% specificity, 75% sensitivity).

**Conclusion:**

Ultrasound measurement of gastric filling shows promise as a valuable screening tool for detecting reduced gastric motor function in patients with stroke.

## 1 Introduction

Research has shown that up to 75% of survivors of stroke experienced some form of disability, with a significant proportion becoming bedridden at some point during their recovery period (Wang et al., 2021). Patients with long-term bedridden stroke face numerous challenges: besides neurological deficits, they also suffer from a wide range of complications, such as gastrointestinal dysfunction, throughout all recovery periods, from hyperacute to chronic, which places a heavy burden on their overall health and greatly slows brain recovery after stroke. Studies have demonstrated that gastrointestinal dysfunction is common among patients with stroke and significantly contributes to increased incidence, recurrence, and mortality rates of stroke (Tuz et al., 2022; Du et al., 2020; Roth et al., 2020; Orthey et al., 2020). Therefore, screening and evaluating the gastric function of patients with stroke who have been bedridden for a long time is essential; however, this has not yet received the attention of clinicians.

Routine screening does not include the examination of gastric motor function. One contributing factor is the absence of suitable examination approaches. Gastric scintigraphy is commonly used and considered the gold standard for assessing gastric motility; however, participants are exposed to significant radiation and frequently produce false-positive results during the liquid phase (Orthey et al., 2020). Gastric filling ultrasonography, which is simple, non-invasive, nonradioactive, and highly reproducible, offers significant clinical advantages. Currently, clinical studies employ this method to assess gastric motor function in patients with conditions such as diabetes, esophageal achalasia, and functional dyspepsia, as well as in pregnant women and children (Nascimento et al., 2019; Zhang et al., 2020; Steinsvik et al., 2020). To date, no clinical trials have used ultrasonography to assess gastric motor function in patients with long-term bedridden stroke. To address this gap, we employed gastric ultrasound to assess gastric motor function (gastric emptying and gastric motility) in these patients and determine its reliability and effectiveness as a screening tool. Additionally, we analyzed the correlation between gastric emptying or motility indicators and the clinical characteristics of patients with stroke. Our goal was to assess whether routine gastric motility screening should be recommended for patients with long-term bedridden stroke.

## 2 Methods

### 2.1 Participants

A total of 37 patients long-term bedridden with stroke admitted to the Rehabilitation Medicine Department of Shenzhen University First Affiliated Hospital between October 2023 and June 2024 were recruited, and 31 healthy controls (HC) matched for age and sex were recruited from their relatives and caregivers. Patients with stroke fulfilled the diagnostic criteria of stroke in the “Chinese Stroke Association guidelines for clinical management of cerebrovascular disorders” (Lou et al., 2020) and were diagnosed with stroke by head computed tomography (CT), magnetic resonance imaging (MRI), and alternative imaging techniques, and bed confinement for ≥30 days. Patients with a history of gastrointestinal diseases (e.g., malignant tumors of the stomach, subtotal gastrectomy, stomach or duodenal ulcer, and reflux esophagitis) or those who required medications known to affect gastrointestinal function (e.g., digitalis, aminophylline, and non-steroidal anti-inflammatory drugs) were excluded. The research protocol was approved by the Ethics Committee of the Shenzhen University First Affiliated Hospital (registration number: 2023-245-02PJ). Before participating, all individuals signed a written informed consent form. In this study, sample size calculations were performed using GPower 3.1 software (Franz Faul, Universität Kiel, Germany). The estimation was based on the repeated measures ANOVA method provided by GPower. A significance level of α = 0.05 was used for a two-tailed test, with a Type II error probability (β) of 0.2 and a statistical power of 0.8. The medium effect size was set to 0.06, with an expected effect value of 0.25.

### 2.2 Clinical data collection

**FIGURE 1:**
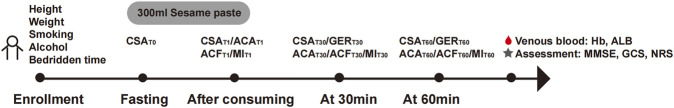
Study protocol. A schematic illustration of the study design depicting the study timeline and study-related activities.

The height, weight, smoking history, and alcohol history of all participants were collected, and each patient was evaluated using the Glasgow Coma Scale (GCS), Mini-Mental State Examination (MMSE), nutritional risk screening (NRS), and albumin (ALB) and hemoglobin (Hb) levels. The protocol used in this study is shown in Figure 1.

### 2.3 Gastric-filling ultrasonography evaluation

**FIGURE 2:**
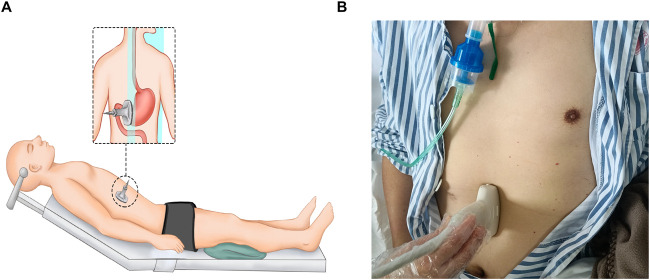
Representative probe placements in supine position.

**FIGURE 3:**
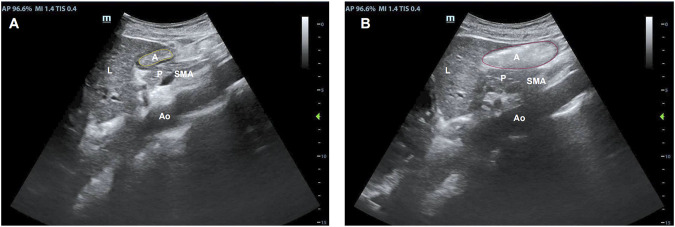
Sonographic identification of the gastric antrum with the aid of anatomical landmarks: A, the gastric antral cross-sectional area; Ao, the abdominal aorta; L, the left lobe of the liver; P, the inferior pancreas; and SAM, the superior mesenteric artery. The gastric antrum cross-sectional area after **(A)** fasting and **(B)** immediately after a meal.

The participants were placed in a supine position with the head of the bed raised 30°–45°. The Mindray M9 portable color Doppler ultrasound diagnostic equipment was used, and a convex array ultrasound probe operating at a frequency of 3–5 MHz was chosen (Figure 2). The gastric antrum section was located below the middle xiphoid process of the upper abdomen using the abdominal aorta, superior mesenteric artery, and left lobe of the liver as markers (Figure 3). After six to eight hours on an empty stomach, healthy participants took 300 mL of sesame paste orally to fill the stomach cavity, while patients bedridden with stroke injected it through a gastric tube to fill the stomach cavity.

### 2.4 Gastric emptying index

The cross-sectional area of the gastric antrum (CSA) is utilized for measuring the gastric emptying rate (GER). As mentioned earlier, after determining the left upper lobe of the liver and the main abdominal arteries, the gastric antrum was located, and its anteroposterior axis diameter (AP) and head-tail diameter (CC) were measured. To calculate the CSA, take the average of three consecutive measurements using the formula: CSA = π × AP × CC/4. The area and diameter of the slurry layer were measured at the following time points: after fasting for six to eight hours (T0), immediately after consuming meal (T1), and 30 min (T30) and 60 min (T60) post-meal. The calculation formula for GER was: (1- CSA at T30/T60)/CSA at T1 × 100% (Lin et al., 2023). All scans were conducted by the same experienced physician, as variations in the force applied to the ultrasound probe could potentially influence cross-sectional interpretation. We obtained ultrasound parameters for each subject in triplicate and conducted a reliability analysis to assess the effectiveness and consistency of ultrasound testing. The calculated ICC was 0.90 (*P* < 0.001), indicating good reliability of ultrasound in detecting CSA in participants.

### 2.5 Gastric motility index (MI)

Gastric motility was assessed by evaluating the contraction movements of the gastric antrum, focusing on indicators such as the amplitude (ACA) and frequency (ACF) of gastric antrum contraction, as well as the MI. The number of antral gastric contractions was continuously recorded within 3 mins of filling, and the number of antral gastric contractions per minute was recorded as the ACF. Then, the area of the maximum relaxation (Sr) and contraction (Sc) of the gastric antrum was continuously measured three times, and the change in gastric antral volume was calculated (ΔS) = Sr-Sc; ACA is calculated as ΔS/Sr, and MI is determined by the formula MI = ACF × ACA (Zou et al., 2024).

### 2.6 Statistical analysis

Demographic and baseline features were presented as N (%) for categorical data and mean (standard deviation) for continuous data. Group comparisons were conducted using a two-tailed Student’s t-test for continuous variables and the chi-square test for categorical variables. The CSA and GER between the case and control groups were compared using repeated-measures analysis of variance. Group comparisons of normally distributed data were conducted using an independent sample t-test between the two groups, whereas comparisons between the case and control groups of non-normally distributed data were conducted using a non-parametric test. The relationships among GER, ACF, ACA, and bed rest duration were determined using Spearman’s rank correlation analysis. The relationship between GER, ACF, ACA, and clinical features (such as cognitive level and nutritional status) of patients with stroke was analyzed through multiple linear regression, and receiver operating characteristic (ROC) curves were used to evaluate the specificity and sensitivity of gastric emptying and gastric motility indicators in predicting clinical symptoms in patients. Using SPSS 23.0 and Graphad Prism 9.0 for analysis, *P* < 0.05 signifies statistical significance.

## 3 Results

### 3.1 Comparison between groups

Between October 2023 and June 2024, 68 participants (37 patients and 31 healthy controls) were recruited. Table 1 presents the demographic and baseline characteristics of patients in each group. There were no significant differences between the stroke and HC groups in terms of sex, age, smoking and drinking history, height, weight, and BMI (*P* > 0.05). The average bed rest of stroke group patients was 3.54 ± 2.46 months, with the longest being 11 months and the shortest being 1 month.

**TABLE 1:** Demographics and baseline characteristics.

Demographics	Stroke patients	Healthy control	*P*
	(n = 37)	(n = 31)	
Male sex	21 (56.76)	17 (54.84)	0.29
Age (years)	65.46 ± 2.15	63.00 ± 2.11	0.42
Smoking	13 (35.14)	9 (29.03)	0.60
Drinking	7 (18.92)	7 (22.58)	0.71
Height (cm)	164.10 ± 1.21	164.40 ± 0.98	0.86
Weight (kg)	57.29 ± 1.61	59.24 ± 1.37	0.37
BMI [kg/m^2^]	21.26 ± 0.52	21.93 ± 0.50	0.35
Lying-bed period (months)	3.54 ± 2.46	NA	NA

The data are presented as mean ± standard deviation or as number (percentage).

### 3.2 Comparison of gastric emptying index between groups

**FIGURE 4:**
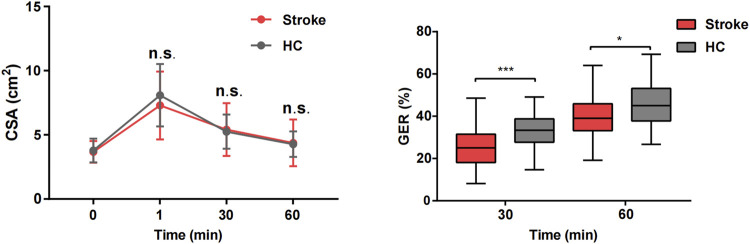
Comparison of gastric emptying index between stroke patients and healthy control groups. *P < 0.05 and ***P < 0.001.

As illustrated in the CSA curve of the gastric antrum (Figure 4), there were no significant differences in the CSA between the stroke and HC groups at T0, T1, T30, and T60 (*P* > 0.05). Compared to the HC group, the GER of the stroke group was slower at T30 (GER30) and T60 (GER60), with a significant decrease in emptying speed from 0 to 30 min (*P* < 0.05).

### 3.3 Comparison of gastric motility index between groups

**FIGURE 5:**
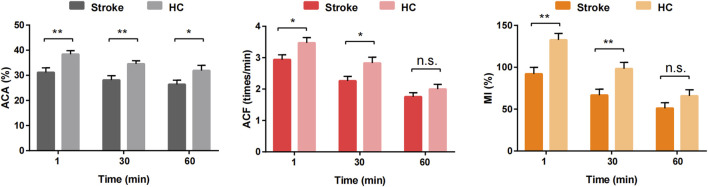
Comparison of gastric antral motility at different time points between stroke patients and healthy control groups. *P < 0.05, **P < 0.01 and n.s. *P* > 0.05.

Gastric motility indices (ACA, ACF, and MI) were significantly lower in the stroke group than in the HC group at both T1 and T30 (*P* < 0.05). However, no significant differences in ACF and MI were observed between the two groups at T60 (*P* > 0.05). Additionally, as illustrated in Figure 5, there was a noticeable decreasing trend in the gastric motility indices over time in both groups. A video comparing the gastric motility between the two groups is shown in Supplementary Material.

### 3.4 Relationship between gastric emptying or motility index and clinical characteristics

Based on the above results, we conducted a correlation analysis between the gastric parameters with statistical differences and the clinical characteristics of the patients (consciousness and cognition status: MMSE/GCS; nutritional status: ALB, HB, and NRS). The results revealed no significant association between gastric emptying or motility indices and cognitive consciousness status, whereas ACA at various time points and GER30 correlated with ALB, and GER30 as well as ACA_T60_ were correlated with HB (Table 2). Spearman rank correlation analysis showed that the ACA at various time points, GER30, GER60, and MI_T1_ was negatively related to the length of bed rest (Table 3).

**TABLE 2:** Multiple linear regression analysis of gastric motility or emptying indicators and nutritional status.

		B	SE	95% CI	P
Dependent Variable: ALB	GER30	1.639	0.140	0.314 to 0.888	0.000
	GER60	−0.078	0.072	−0.177 to 0.119	0.689
	ACF_T1_	−0.711	2.531	−8.171 to 2.215	0.250
	ACF_T30_	0.558	2.209	−2.038 to 7.028	0.269
	MI_T1_	−0.503	0.068	−0.181 to 0.099	0.552
	MI_T30_	0.363	0.065	−0.100 to 0.167	0.615
	ACA _T1_	−1.464	0.250	−1.037 to 0.010	0.046
	ACA _T30_	−1.439	0.240	−1.027 to 0.041	0.035
	ACA _T60_	1.786	0.233	0.205 to 1.162	0.007
Dependent Variable: HB	GER30	0.689	0.388	0.197 to 1.790	0.016
	GER60	0.045	0.200	−0.345 to 0.476	0.745
	ACF_T1_	0.480	7.021	−6.509 to 22.305	0.271
	ACF_T30_	0.513	6.129	−3.555 to 21.597	0.153
	MI_T1_	−0.726	0.190	−0.623 to 0.155	0.228
	MI_T30_	−0.221	0.181	−0.449 to 0.291	0.665
	ACA _T1_	−0.442	0.694	−2.045 to 0.804	0.379
	ACA _T30_	−0.750	0.667	−2.462 to 0.275	0.113
	ACA _T60_	1.673	0.647	1.189 to 3.845	0.001

**TABLE 3:** Spearman rank correlation analysis between gastric motility or emptying indicators and bed rest duration.

Index	Correlation coefficient	P
GER_T30_	−0.496	0.002**
GER_T60_	−0.368	0.025*
ACF_T1_	0.048	0.779
ACF_T30_	0.041	0.809
MI_T1_	−0.329	0.047*
MI_T30_	−0.184	0.277
ACA _T1_	−0.518	0.001**
ACA _T30_	−0.361	0.028*
ACA _T60_	−0.329	0.047*

*P < 0.05, **P < 0.01.

**FIGURE 6:**
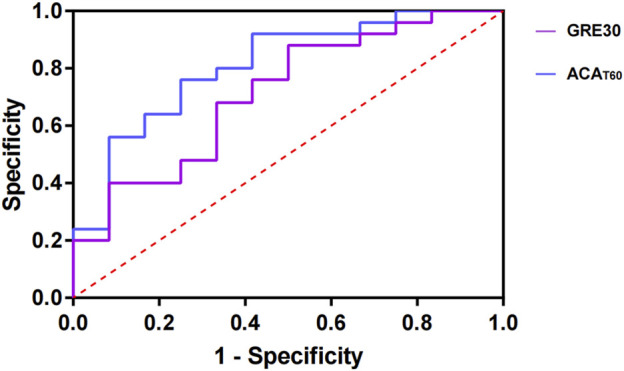
ROC curve of GER30 and ACA_T60_ for predicting anemia status in stroke patients.

The feasibility analysis of ACA or GER30 in determining hypoalbuminemia (ALB < 35 g/L) and of GER30 or ACA_T60_ in diagnosing anemia (HB < 110 g/L) revealed that ACA and GER30 were not effective predictors of hypoalbuminemia, whereas ACA_T60_ and GER30 could, to some extent, determine the anemia status in patients. According to the analysis of the area under the ROC curve, when the optimal cut-off value of GER30 was 31.52, the specificity was 88%, but the sensitivity was relatively low at 50%. The area under the curve (AUC) for GER30 was 0.71, with a *P* value of 0.038. For ACA_T60_, when the optimal cutoff value was 23.64, the sensitivity was 75%, and the specificity was 76%, with an AUC of 0.81 and a *P* value of 0.003 (Figure 6).

## 4 Discussion

Our results confirm that patients with stroke with a long-term bedridden status experience delayed gastric emptying and decreased gastric motility, which is consistent with previous studies (Camara-Lemarroy et al., 2014; Yong et al., 2023). In recent years, research on the mechanisms of gastrointestinal dysfunction in stroke has mostly focused on the influence of the brain-gut axis, vagus nerve damage, inflammatory mechanisms, and gut microbiota (Tuz et al., 2022; Benakis and Liesz, 2022; Zhang et al., 2024). Gastrointestinal complications occur in more than half of survivors of stroke and are believed to lead to neurological outcomes of stroke dysfunction (Yong et al., 2023). Our results also indicated that various stomach parameters showed a decreasing trend over time after meals, and the GER, ACF, ACA, and MI changed most significantly within 30 min after meals, whereas only ACA differed from normal participants 60 min after meals. However, the reason for this is unclear and further investigation is required. This suggests that gastric dysfunction in patients with stroke mainly occurs within half an hour after meals and is reflected in the amplitude of gastric contractions rather than the frequency of contractions. Under normal circumstances, a certain contraction frequency, accompanied by an appropriate contraction amplitude, can ensure the effective emptying of food from the stomach. However, this coordination may be disrupted under pathological conditions. For example, in patients with functional dyspepsia, although the contraction frequency increases, the contraction amplitude is insufficient, resulting in low emptying efficiency (Wang et al., 2023). Therefore, it is speculated that gastrointestinal dysfunction in patients with long-term bedridden stroke may also be characterized by an imbalance in the coordination between the ACA and ACF. The 300 mL homogenate used in this study can usually be emptied within approximately 30 min in normal individuals (Farrell, 2024), which may explain why the difference in the emptying index between patients with stroke and normal individuals is more pronounced at 30 min.

To further explore the potential pathophysiological mechanisms underlying these findings, we propose that the brain-gut axis plays a critical role in mediating gastric motility changes in stroke patients. Specifically, the disruption of neural pathways, such as vagus nerve dysfunction, may impair the normal regulation of gastric contractions and emptying (Décarie-Spain et al., 2024). In stroke patients, damage to brain regions that control this axis—such as the brainstem—could lead to impaired regulation of gastric contractions, contributing to delayed gastric emptying and abnormal motility patterns. Additionally, neuroendocrine dysregulation, including alterations in stress hormones like cortisol and catecholamines, could further exacerbate gastrointestinal dysfunction by affecting the contractile properties of gastric smooth muscle (Longo et al., 2023). The inflammatory response post-stroke, characterized by the release of pro-inflammatory cytokines, may also contribute to gastric dysmotility by inducing local inflammation in the gastrointestinal tract (Agirman et al., 2021). Furthermore, changes in gut microbiota composition, often observed in stroke patients, could influence gastric motility through the production of metabolites that affect neural and hormonal signaling (Aburto and Cryan, 2024). These mechanisms collectively suggest that the observed gastric dysfunction in stroke patients is multifactorial, involving complex interactions between the central nervous system, neuroendocrine pathways, and the gastrointestinal environment. However, our study suggests that while these mechanisms are likely involved, further exploration of how they specifically relate to the results observed in stroke patients with long-term bedridden status is needed.

Additionally, a long-term bedridden status is a risk factor for gastrointestinal dysfunction. Our study found that gastric emptying and motility indicators (GER30, GER60, MI_T1,_ and ACA at various time points) were related to bed rest time. The longer the bed rest time, the worse the gastric emptying and motility parameters, which is consistent with the findings of previous studies (Tuz et al., 2022). Therefore, some researchers have proposed that early bedside bicycle exercises can improve gastrointestinal function in patients who are bedridden (Yu et al., 2022).

Another important finding of this study is that certain gastric-related ultrasound parameters (GER30, ACA, and MI_T1_) are associated with the nutritional status of patients with stroke, among which ACA_T60_ and GER30 can predict anemia to some extent. Insufficient gastric motility (such as delayed gastric emptying) can affect the processing time of food in the stomach, thereby affecting the absorption of nutrients (including iron and vitamin B12, etc.). These nutrients are crucial for the generation of red blood cells (Bloor et al., 2021; Stein et al., 2016).

Previous studies have indicated that scintigraphy is the gold standard for assessing gastric function; however, it has the drawback of radiation exposure. Wireless smart capsule pills offer a radiation-free alternative, but their validation rate falls short compared with scintigraphy and are unsuitable for patients with pacemakers or defibrillators (Lee et al., 2019). Although the measurement of gastric electrical activity has a certain application value in evaluating gastric motility disorders, it also has drawbacks, such as signal interference and the inability to evaluate the mechanical contraction function of the stomach in detail by measuring only the slow-wave electrical activity (Wang et al., 2023). Recent advancements in MRI technology have introduced new methods for non-invasive evaluation of gastric function. One notable innovation is developing a 3D stomach model derived from MRI that offers detailed assessment capabilities for surface geometry, gastric volume, and wall tension (Bertoli et al., 2023). MRI might offer greater accuracy but is limited by its high cost, complexity, time requirements, and certain methodological constraints, rendering it unsuitable for routine clinical screening of gastric motility impairment in patients with stroke and those requiring dynamic monitoring of gastric function.

Among the existing methods of gastric evaluation, gastric ultrasonography has emerged as a safe, effective, and convenient approach for assessing gastric function. Its advantages include being non-invasive and non-radiative, allowing real-time dynamic observation, ease of operation, and repeatability. It can safely and quickly assess the anatomical structure and functional status of the stomach, including the measurement of gastric emptying time and evaluation of gastric wall movement. This method is particularly suitable for patient populations requiring frequent monitoring. In 1980, Holt et al. pioneered the use of ultrasound to examine gastric contractions in healthy individuals after a liquid test meal (Holt et al., 1980). Since then, the use of ultrasonography for detecting gastric peristalsis has increased significantly. Currently, ultrasound is extensively utilized across various medical fields to screen for gastric function disorders, and its accuracy is well-established and validated (Lyons and El-Boghdadly, 2024; Kruisselbrink et al., 2019; Muresan et al., 2015; Bouvet et al., 2020). This study showed that gastric-filling ultrasound examination is feasible for determining the gastrointestinal dynamics of patients with stroke who have been bedridden for a long time.

Our study had some limitations. First, as a case-control study, it does not allow for prospective observation of changes in gastric motor function in patients with stroke. Second, ultrasound relies heavily on the operator’s skill and has a significant learning curve, necessitating all examinations to be conducted by the same individual. Additionally, the limited sample size may impact the generalizability and stability of the findings, as the results might not fully represent the broader stroke population with varying disease types, severity, lesion sites and stages. In future studies, we recommend expanding the sample size and including a more diverse cohort of stroke patients to enhance the reliability and applicability of the conclusions.

## 5 Conclusion

In summary, patients with stroke who are bedridden for extended periods experience delayed gastric emptying and reduced gastric motility, along with impaired coordination of gastric contraction amplitude and frequency. Gastric ultrasound indicators are closely related to the nutritional status of patients and can predict the presence of anemia. Gastric ultrasound examination is straightforward, reproducible, nonradioactive, and precise. Clinically, it can be routinely employed to identify gastric motility disorders and monitor changes in gastric motor function in patients with bedridden stroke.

## Data Availability

The original contributions presented in the study are included in the article/Supplementary Material, further inquiries can be directed to the corresponding author.
